# Patient and Provider Perspectives on Symptom Monitoring During Outpatient Chemotherapy: Interview Study

**DOI:** 10.2196/46001

**Published:** 2023-04-17

**Authors:** Leeann Chen, Christianna Bartel, Xinlu Cai, Yanghuidi Cheng, Adam Perer, Sean McClaine, Elizabeth Kairis, Krina Durica, Weiyu Huang, Carissa A Low

**Affiliations:** 1 Department of Medicine University of Pittsburgh Pittsburgh, PA United States; 2 Human-Computer Interaction Institute Carnegie Mellon University Pittsburgh, PA United States

**Keywords:** symptom management, remote patient monitoring, doctor-patient communication, mobile technology, cancer, ambulatory, chemotherapy, oncology, remote monitoring, outpatient, mobile phone

## Abstract

**Background:**

Fluctuating symptoms and side effects are common during outpatient cancer treatment, and approaches to monitoring symptoms vary widely across providers, patients, and clinical settings. To design a remote symptom monitoring system that patients and providers find to be useful, it may be helpful to understand current clinical approaches to monitoring and managing chemotherapy-related symptoms among patients and providers and assess how more frequent and systematic assessment and sharing of data could improve patient and provider experiences.

**Objective:**

The goals of this study were to learn about patient and provider perspectives on monitoring symptoms during chemotherapy, understand barriers and challenges to effective symptom monitoring at one institution, and explore the potential value of remote symptom monitoring between provider visits.

**Methods:**

A total of 15 patients who were currently undergoing or had recently completed chemotherapy and 7 oncology providers participated in semistructured interviews. Interviews were transcribed and coded using an iterative thematic analysis approach. The study was conducted at a National Cancer Institute–Designated Comprehensive Cancer Center.

**Results:**

Four main themes were discussed by patients and providers: (1) asynchronous nature of current methods for tracking and managing symptoms, (2) variability in reported symptoms due to patient factors, (3) limitations of existing communication channels, and (4) potential value of real-time remote symptom monitoring during chemotherapy. Current asynchronous methods and existing communication channels resulted in a disconnect between when symptoms are most severe and when conversations about symptoms happen, a situation further complicated by memory impairments during chemotherapy. Patients and providers both highlighted improvements in patient-provider communication as a potential benefit of remote real-time symptom monitoring. Providers also emphasized the value of temporal data regarding when symptoms first emerge and how they progress over time, as well as the potential value of concurrent activity or other data about daily activities and functioning. Patients noted that symptom monitoring could result in better preparation for subsequent treatment cycles.

**Conclusions:**

Both patients and providers highlighted significant challenges of asynchronous, patient-initiated, phone-dependent symptom monitoring and management. Oncology patients and providers reported that more routine remote monitoring of symptoms between visits could improve patient-provider communication, prepare patients for subsequent chemotherapy cycles, and facilitate provider insight and clinical decision-making with regard to symptom management.

## Introduction

### Background

Chemotherapy can cause a variety of symptoms and side effects that can impair patient quality of life and functioning [1,2]. Unfortunately, symptoms remain undetected by cancer clinicians up to half the time [3,4], limiting opportunities for effective clinical management. This may be in part due to current approaches to symptom monitoring relying heavily on assessing symptoms at outpatient clinic visits, while severe side effects and toxicities usually occur between these clinical encounters. When symptoms emerge or worsen between visits, detection of these issues relies on a patient’s ability to determine which symptoms require clinical attention and to access necessary care, the discernment of which can be difficult at times [5].

Improving clinical approaches to monitoring symptoms during chemotherapy could improve clinician capture and manage severe symptoms, reduce symptom burden and unplanned health care use, and improve patient quality of life and outcomes. Routinely collecting patient-reported assessments of symptom severity, frequency, and interference could result in more thorough communication of subjective symptoms to clinicians and improved patient-provider communication. With the growing ubiquity of smartphones and patient portals, a number of systems have leveraged technology for patient-reported symptom monitoring during chemotherapy [6-9]. These electronic symptom self-reporting systems vary in how often they assess symptoms, what instruments are used to assess symptoms, and what follow-up with providers or other features are available, but they have been shown to be generally acceptable and feasible [10-12].

To design a remote symptom monitoring system that patients and providers find to be useful, it may be helpful to understand current clinical workflows and approaches to monitoring and managing chemotherapy-related symptoms from both the patient and provider perspectives and how more frequent and systematic assessment and sharing of data could improve patient and provider experiences.

### Objective

The purpose of this study was to learn about patient and provider perspectives on monitoring symptoms during chemotherapy, understand barriers and challenges to effective symptom monitoring at one institution, and explore the potential value of remote symptom monitoring between provider visits.

## Methods

### Ethics Approval

This study was conducted at a National Cancer Institute–Designated Comprehensive Cancer Center. The Institutional Review Board of the University of Pittsburgh approved all study activities (STUDY21070181). Data collection was conducted between December 2021 and August 2022.

### Participants

#### Patients

We recruited patients who were currently undergoing or had undergone chemotherapy within the past year from 2 sources: participants who were enrolled in or had recently completed an ongoing study assessing daily symptoms and activity (n=9) and a community research registry (n=6). Patients were eligible if they were (1) currently undergoing or had completed chemotherapy for cancer within the past year, (2) aged 18 years or older, and (3) fluent in English. Patients received US $50 in compensation for their participation.

#### Providers

A convenience sample of oncology providers (n=7) was recruited through purposive sampling. Providers were eligible if they were (1) currently working as a clinical oncology provider caring for patients receiving treatment for cancer, (2) aged 18 years or older, and (3) fluent in English. Providers also received US $50 in compensation for their participation.

#### Procedure

Semistructured interview guides were developed by the research team for each population, with a focus on open-ended questions to elicit participant experiences with symptom monitoring. Interviews lasted between 30 and 90 minutes and were conducted remotely over Zoom or in-person by members of the research team. Interviews were digitally recorded, transcribed, deidentified, and entered into the qualitative analysis software, Dovetail.

#### Data Analysis

The research team analyzed transcripts using an iterative thematic analysis approach [13]. A coding team comprised of 3 authors read all transcripts and developed preliminary codes. All transcripts were coded by a member of the coding team, with weekly meetings to ensure consistency of coding and group codes into larger thematic classifications.

## Results

### Participant Characteristics

Patients (mean 51, SD 19.2, range 19-82 years; 10/15, 67% White; 6/15, 40% female) had a variety of primary diagnoses, including pancreatic cancer (n=3), colorectal cancer (n=3), breast cancer (n=2), other solid tumors (n=2), and leukemia or lymphoma (n=5). Most patients (10/15, 67%) had attended at least some college.

### Providers

Providers included physicians (n=3), advanced practice providers (n=3), and a registered nurse (n=1) who had a mean age of 50 (SD 10.2; range 29-59) years were primarily White (5/7, 71%) and female (5/7, 71%) and had been treating patients with cancer for an average of 8.6 (SD 3.82; range 4.5-16) years.

### Themes

Four primary themes were discussed by patients and providers: (1) asynchronous nature of current methods for tracking and managing symptoms, (2) variability in reported symptoms due to patient factors, (3) limitations of existing communication channels, and (4) potential value of real-time remote symptom monitoring during chemotherapy.

#### Theme 1: Asynchronous Nature of Current Methods to Track and Manage Symptoms

Providers and patients described current approaches to tracking symptoms as largely asynchronous. Both patients and providers mentioned that prior to the first treatment cycle providers gave written or verbal information about what symptoms to expect during chemotherapy, how to manage them, and when to reach out to providers. Regarding his cancer journey, patient 22 emphasized the overwhelming information provided in preparation for treatment:

You’re given a lot of papers to read… pretty much like what the drug is, what side effects you'll get, you know, and just reading all of that, then of course, unfortunately, the internet which has everything on everything you could possibly be affected by these chemo drugs, you know. All that was a lot to comprehend and digest. I think that digest is maybe a better word. Yeah, I think that part of the journey was difficult, you know, it was difficult, and I'd almost gotten to the point where I wasn't going to do chemotherapy.

Importantly, this information is provided at a time when patients were already overwhelmed by their recent diagnosis and treatment decisions, and often before patients have begun to experience any of the potential side effects. This may make it difficult for patients to remember or find the information they need when they begin experiencing symptoms.

Both groups also stated that although providers ask about common symptoms at scheduled visits, patients are expected to call providers between visits as needed. They expressed a disconnect between when symptoms were most severe and when providers would follow up on calls or when conversations about symptoms happened during appointments. Patient 20 remarked:

Sometimes when I had nausea and I didn't know like what nausea medicine to take or something I would call there, and they just didn't answer and then, like, I don't know, a couple like, maybe like a week later they’d answer, but my symptom’s already gone by then because after treatment’s the worst and by the time they got back to me, I was better.

Some patients (n=7) described developing their own system for tracking symptoms and other relevant health information. Patient 4 sought out medical literature regarding his specific cancer, which inspired him to log his symptoms daily according to the Bristol Stool Chart [14], in addition to manually transcribing clinical lab values and test results into a spreadsheet. Patient 6 periodically used a blood pressure cuff and a mobile app with an atrial fibrillation monitor. Others used physical journals, notes apps on their mobile phone, word processing documents, or scraps of paper, citing convenience and flexibility of the interface to accommodate their needs as reasons for their tracking method. Patients who described a tracking system mentioned that they did not share these data directly with their providers but did refer to their notes or system as needed when talking with providers. Consistent with this, Provider 19 observed:

I have many patients who have pieces of note papers, they’re all over the place, they’re kind of like scribbled on.

#### Theme 2: Variability in Reported Symptoms Due to Patient Factors

The asynchronous approach to symptom monitoring meant that patients were often responsible for bringing up symptoms during clinical appointments or calling providers between visits to report severe symptoms. Both patients and providers mentioned that symptoms were often underreported, reported to be less bothersome or severe, or not discussed even though they impacted quality of life. From the providers’ point of view, they reasoned that patients worry their treatment may be withheld or reduced if they complain of symptoms and that patients do not want to bother clinicians between visits. Provider 17 stated:

And then, what I always tell my patients is really the best treatment is the kind of treatment that you can handle and you can still live a good life. So I always try to reinforce to patients that but some of them get kind of scared that if they bring up a complaint you're going to mess with a dose and it's not going to be as good, and I think that's always that's an ongoing battle with some patients.

Of note, no patients explicitly voiced concerns during study interviews that treatments would be reduced or withheld if they reported symptoms.

Both groups also acknowledged that some patients believe they should “tough it out” and that side effects are a part of chemotherapy that they need to accept. When asked why Patient 5 did not track her symptoms, she replied:

I didn't track them. I didn't. I did not track anything, I mean, I just. All I would tell you is. I am- I am a tough person. I just move on. Just keep moving on.

Provider 17 also noted:

Some patients kind of try to tough it out a little bit too much, you know. And then you see them and they’re, they’ll be like, I’ve been throwing up and having diarrhea for two weeks...and that’s always kind of sad to hear because I always tell the patients to let us know if that’s happening before I see you again, because you shouldn’t be suffering for two weeks.

In a similar vein, provider 19 further elaborated on how patients who minimize their symptoms need to be probed symptom by symptom to get a better idea of their well-being:

Because some of our patients are notorious for every time you ask them how they're feeling they're like ‘oh, I'm like, I feel fine’, but then like once you start to specifically ask them it's like are you, you know, are you having any nausea, are you having any diarrhea, once you start to ask them specific questions and then they'll be like ‘oh yeah, well there was, you know, the other day, like I threw up my entire dinner’. So sometimes you kind of, some patients you need to really direct them, you need to ask them very specific questions about specific symptoms, because if you just ask them overall like, you know, how things are going, some of them are just like, like ‘it's okay, it's fine.’

Providers also remarked that having additional information about activities and functioning could help them better understand how a patient’s symptoms were interfering with their lives. Provider 13 noted:

Some people just kind of suffer quietly so you have to kind of drag, you know, kind of investigate...what’s going on at home, what can they do, can they fold laundry, are they doing dishes, can they sit on their front porch and talk to their neighbors.

Provider 18 said:

One thing I tell people, which I found helpful, is if you find that you can’t do the things that you were doing three weeks ago because of any symptom, you don’t have to figure out why it is, you just have to call us.

These quotes reflect how providers probe for activities of daily living and instrumental activities of daily living to assess whether and how symptoms are interfering with functioning and quality of life.

Furthermore, patients and providers described examples of inaccurate reporting because *symptoms themselves can interfere with reporting.* Memory problems were the most commonly cited symptom to interfere and made it particularly difficult for patients to wait until their doctor’s appointment to bring up symptoms. Patient 7 remarked:

They asked, like I said, they try to ask you, you know, what's going on. Is the nausea bad, this and that but, like I said you're getting this whitewashed version, because I forgot what happened 10 days ago.

Patient 2 noted:

I really should have written more down because chemo brain is very real and you’d forget.

#### Theme 3: Limitations of Existing Communication Channels

Providers in particular noted several limitations of existing channels for patients to communicate changes in symptoms to their care team and providers to assess and manage symptoms. First, providers noted that *high patient volume limits their face time with each patient,* which constrains discussion of symptoms during scheduled clinic visits.

Second, providers and patients mentioned that *relying on the phone as the primary mode of communication was inefficient.* Provider 10 reported that the clinic depends on a 24/7 helpline for patients to report symptoms and uses a combination of phone and email but has had trouble with patients triaging their concerns with the appropriate tool. Meanwhile, provider 19, a nurse, pointed to the burden of *playing phone tag* with patients as the initial point of contact. Finally, provider 16 discussed the challenges around understanding what might be going on with a patient when filtered through multiple messages and informants:

If they’re going to call with symptoms, they’re going to call my nurse, and so my nurse will triage. You know they’ll be like, hey, so-and-so called, they’re complaining of xyz, and my nurses are great but already that’s like, already it’s a step removed, like I wasn’t the one who talked to the patient. You know, sometimes it’s obvious you know what to do, what not to do, but other times I’m like, I probably need more information about this. So oftentimes, like, I have to still call the patient myself.

This comment reflects the challenges of aligning an interdisciplinary clinical team around sporadic, subjective data communicated via telephone.

#### Theme 4: Potential Value of Real-time, Standardized Remote Symptom Monitoring During Chemotherapy

Finally, participants in both groups discussed the potential value of real-time remote symptom monitoring during chemotherapy. The most common belief shared by patients and providers was that real-time remote monitoring could *improve patient-provider communication.* Patient 2 acknowledged her tendency to underreport and how having her data on hand would help her be more aware of her symptoms so she could bring them up to her doctor:

I don't know, being more aware of them definitely. Like I said there's some things that for me it's not worth telling the doctor, but when I go and actually look at them it's like ‘oh yeah, that whole week it really was an issue.’ Maybe I should have said something.

In a similar vein, Provider 16 believed that data from wearable activity monitors or similar devices could improve patient-provider communication because it provided more objective data:

In terms of things that patients can inaccurately report or variably report, like activity level is like one of the hardest ones to tease out, you know, and so I like that this is a more objective measure of what the patient's actually doing.

Provider 17 then gave an example of how he would incorporate step count from continuous monitoring into communicating with his patients about managing treatment goals and expectations:

That's a situation where I honestly tell most, almost all patients that it's time to focus really just on symptom management and some of this sometimes being able to say look this is where we were a couple months ago, you were taking, you know, 5000 steps a day you had zero fatigue blah blah, and now this is where we are now. You know, I think what you’re telling us is you aren’t doing as well as we would have wanted. Maybe we should sit down and talk more about, you know, where we are with treatment and whether or not we’re accomplishing our goals with chemotherapy.

Among provider interviews, there was also unanimous consensus that *temporal information collected about precisely when symptoms emerged and unfolded over time would be useful to them in gaining insight* into patient symptom causes and consequences and prescribing a more precise and timely symptom management strategy. Provider 18 explained:

If a patient were to log their pain scale, for example, every day, you could tease out if over time it’s improving because of treatment or if treatment is actually causing them some pain.

Provider 15 noted:

If you know what days they’re on chemo and then you see that there’s constipation, you know, those days and a couple of days after, I mean, this would be really helpful in kind of guiding what days you would want them to take certain medications and things.

Providers also noted that this fine-grained information may allow them to detect issues earlier. Provider 19 stated:

If we know how a patient’s feeling and they can keep us up to date with what’s going on, a lot of times we’re able to help prevent them from having to have a hospital admission or, you know, need to be evaluated in the emergency department.

Finally, the third potential value mentioned by both participant groups was that *continuous remote monitoring could help patients know what symptoms to expect with subsequent treatment cycles and to better prepare for them*. Patient 9 noted:

If you know what's coming, then it's not as scary when it comes.

Likewise, Provider 15 explained how finding patterns in tracked symptoms can help patients cope with the ambiguity of chemotherapy by predicting specific symptoms:

Honestly, I think it would help the patients to kind of see a pattern. You know, okay, I did have decreased appetite these two days this month, and I'm starting chemo here. So you know, do I have it these two or three days again. Okay, maybe it's, this is not totally unexpected, I had the same thing last month and I got through it by you know day three or so.

## Discussion

### Principal Findings

The goal of this study was to better understand oncology patient and provider perspectives on their current symptom monitoring approaches during chemotherapy as well as the potential value of routine and remote symptom monitoring between visits. Both patients and providers considered the current asynchronous nature of symptom monitoring at outpatient clinic visits to be challenging, recognized the variability in reported symptoms due to patient factors, and explained how existing communication channels, such as phone and email, were limited. Some variability in reported symptoms was due to patients believing they should “tough it out,” which may be similar to patients with other chronic illnesses. Both groups saw potential value in real time, standardized, remote symptom monitoring during chemotherapy because of how it could better facilitate patient communication with providers and enable patients to know what symptoms to expect with subsequent treatment cycles so that they and their support systems can better prepare for them. Additionally, clinicians emphasized the need for temporal data regarding when symptoms first emerge and how they progress over time in the context of treatment dates, so providers could prescribe a more precise symptom management strategy.

### Comparison to Prior Work

These results are consistent with earlier qualitative findings highlighting unmet patient needs around symptom management and supportive cancer care [15]. They also echo previous patient-reported advantages (eg, increased self-awareness and improved communication with providers) and disadvantages (eg, belief that symptoms are part of cancer and should be accepted) of symptom monitoring during cancer treatment [16,17]. In addition to confirming these previously reported benefits, providers in this study also noted that more fine-grained temporal information about symptom timing and patterns, especially when combined with information about activity or functioning, could improve their understanding of how to best manage a patient’s symptoms. Our results about the patient- and provider-reported benefits of symptom monitoring also complement emerging evidence that routine symptom monitoring can improve quality of life, reduce health care use, and improve survival [6,18] but that implementation challenges may limit clinical benefits [9].

Taken together, this body of work emphasizes the need to build a remote symptom monitoring system that prioritizes accessibility and leverages the flexible nature of technology to adapt to different user needs. Accessibility in this case should be defined as designing and developing technology in such a way that anyone, regardless of physical or cognitive ability, can use the product. As memory problems were the most-cited symptom that interfered with reporting, patients with cognitive impairments may especially benefit from technology-supported systems with frequent reminders to complete symptom surveys or do other actions like take medications. Given that chemotherapy can also cause peripheral neurotoxicity that impairs fine motor skills and that many patients with cancer are older adults with associated sensory and cognitive impairments as well as lower digital literacy than younger groups, accessibility is an important consideration when designing cancer symptom monitoring and management systems.

### Strengths and Limitations

This study should be evaluated in the context of key limitations. First, convenience sampling of a relatively small group of clinicians was conducted at a single academic cancer center, limiting generalizability. While we aimed to recruit providers in a variety of clinical roles, only 1 registered nurse was interviewed, which may bias clinical perspectives to roles that are more removed from day-to-day patient communication around symptom management. Of note, the electronic medical record system used by many providers within this medical center lacks a patient portal, which could improve some of the communication issues patients and providers noted during interviews. We enrolled patients with diverse cancer diagnoses and stages who were undergoing or had completed any chemotherapy within the course of 1 year, resulting in significant heterogeneity in our sample; focusing on patients with a single type of cancer or receiving more uniform treatment may have yielded different insights. We also recruited patients both from a web-based research registry as well as from an ongoing study assessing daily symptoms during chemotherapy, and patients who had already participated or enrolled in this research may have had greater awareness of their symptoms and side effects. Strengths of the study include the consideration of both patient and provider perspectives, the demographic and medical diversity of our sample, and assessment of both positive and negative aspects of symptom monitoring.

### Future Directions

Findings suggest clinical value of routine symptom and activity monitoring during chemotherapy. Future studies can explore the features of specific technologies such as smartphones and patient portals and their impact on remote patient monitoring. Studies suggest that the interactivity of smartphone apps has made patients feel more involved in their own care and to make symptom reporting relatively easy to integrate into daily routines [19,20]. Mobile or web apps for symptom monitoring could also include dynamic patient-reporting systems where symptom surveys adapt based on specific treatment plans and previously reported symptoms [21]. They could also deliver symptom self-management advice or telehealth visits initiated based on reported symptoms. Branching logic could be used so that if a symptom is reported, the severity, frequency, and impact of that symptom as well as other information that may be useful in determining the best management strategy are assessed. Both physical and psychological symptoms may emerge during outpatient cancer treatment, and remote symptom monitoring could also be leveraged for psychological distress screening during and after cancer treatment [22]. Additionally, future studies can explore how family caregivers can assist in identifying and reporting symptoms as well as potential benefits that remote monitoring might have for caregivers.

These systems should be designed in collaboration with clinicians to determine which symptoms are expected with specific treatments and which symptoms require prompt clinical action. With the recent increased strains on the health care system, clinical staff face the challenge of higher patient volumes. As time becomes more limited to discuss symptoms, personalized symptom management advice integrated into symptom-reporting systems may support patients in addressing their concerns in a timelier manner. To better support patient-provider communication, symptom reporting should also be paired with a well-designed provider dashboard so that all members of the care team as well as patients and their caregivers can view the same real-time information, as well as an alert system to alleviate the patient burden of deciding when their symptoms merit concern and initiating contact as needed. Moving forward, it could be worthwhile to consider follow-up evaluation of symptom management efforts to better understand which actions were successful. Although most oncology providers have experience and expertise in managing symptoms, digitizing symptom monitoring could further allow opportunities for pooled symptom management resources across clinical sites.

In conclusion, both patients and providers highlighted significant limitations of asynchronous, patient-initiated, phone-dependent symptom monitoring and management. Oncology patients and providers reported that more routine remote monitoring of symptoms between visits could improve patient-provider communication, prepare patients for subsequent chemotherapy cycles, and facilitate provider insight and clinical decision-making with regard to symptom management. As more health care systems develop and deploy systems to meet these needs, ensuring that they are implemented in an accessible and equitable way that improves both health care costs and outcomes as well as patient and provider experiences will be critical.

## Data Availability

Data generated and analyzed during this study are not publicly available to protect patient health information but are available as deidentified data from the corresponding author on reasonable request.
